# Recurrent parathyromatosis in a patient with concomitant MEN1 and CASR gene alterations: Clinical management of a case report and literature review

**DOI:** 10.3389/fendo.2023.1108278

**Published:** 2023-03-14

**Authors:** Giulia Sapuppo, Maria Ausilia Giusti, Demetrio Aricò, Romilda Masucci, Martina Tavarelli, Marco Russo, Gabriella Pellegriti

**Affiliations:** ^1^ Department of Clinical and Experimental Medicine, University of Catania, Garibaldi-Nesima Medical Center, Catania, Italy; ^2^ Department of Nuclear Medicine, Humanitas Oncological Centre of Catania, Catania, Italy; ^3^ Clinical and Diagnostic Center, Giovan Battista (G.B.) Morgagni, Catania, Italy; ^4^ Department of Clinical and Experimental Medicine, University of Catania, Catania, Italy

**Keywords:** case report, MEN 1 syndrome, parathyromatosis, CASR alterations, somatostatin analogues (SSAs)

## Abstract

**Introduction:**

Parathyromatosis is a rare cause of primitive hyperparathyroidism characterized by the presence of numerous parathyroid tissue foci in the neck/mediastinum, due to hyperplasia of parathyroid embryologic residues (primary-form) or to local parathyroid tissue implantation (secondary-form). 63 cases have been described in the literature. In our patient parathyromatosis was due to a combination of two mutations.

**Case report:**

A 36-years-old woman was diagnosed with osteoporosis secondary to primary hyperparathyroidism. Subsequent right parathyroidectomy showed a parathyroid adenoma. The follow-up was negative but after 10 years she had a relapse. The genetic screening showed a rare intronic mutation of the MEN1 gene and a heterozygous mutation never described in exon 8 of the CASR gene, coding for the calcium receptor. Calcemia and PTH increased over the years with the onset of nephrocalcinosis and the worsening of osteoporosis despite the therapy with Cinacalcet, bisphosphonates and Vitamin D. She had therefore two additional surgical procedures (parathyroid tissue without malignancy). At follow-up she showed elevated levels of PTH (>1000 pg/ml) and calcium (11.2 mg/dl) and CT scans multiple subcentimetric nodules in the neck/upper mediastinum. Since the ^68^Ga-DOTATATE showed an increased uptake in the neck/mediastinum, lanreotide was added. After two months there was a significant biochemical response but, unfortunately, after six months, the patient showed a new worsening.

**Conclusions:**

a rare case of parathyromatosis due to a combination of two genetic alterations never described. The main issues concern the diagnosis and the radical treatment. Somatostatin analogues may have a useful role in both diagnosis and therapy.

## Introduction

Hyperparathyroidism (HPT) is a rare condition characterized by excessive secretion of PTH. It may be the result of: 1) autonomous secretion of PTH by parathyroid glands (primary hyperparathyroidism, PHPT); 2) excessive secretion of hormone as a consequence of chronic hypocalcemia in patients with chronic renal failure (secondary hyperparathyroidism, SHPT); 3) functional autonomy of parathyroid glands occurs during a long-lasting secondary hyperparathyroidism (tertiary hyperparathyroidism, THPT) (1, 2). PHPT is currently the most common cause of hypercalcemia. PHPT is more frequent in in the postmenopausal years with women/men ratio of 4:1. In Europe, the prevalence is 1.07% (1, 3).

About 90% of all cases are sporadic and 10% are part of a hereditary syndrome, mainly multiple endocrine neoplasia syndromes (MEN-1, MEN-2,MEN-4),hyperparathyroidism-jaw tumor syndrome (HPT-JT), familial hypocalciuric hypercalcemia (FHH-1, FHH-2, FHH-3), familial hypercalciuric hypercalcemia and isolated familial HPT. Hereditary syndromes are characterized by a multiglandular disorder which are asynchronous; the diagnosis is usually defined at early age, generally at the age of 50 years old (1, 4). Almost all patients with PHPT have benign tumors, typical or atypical adenomas (85% of cases) or hyperplasia or multiple abnormal parathyroid glands (15% of cases)). In only 4% of patients are identified Less common causes double adenomas; a very rare cause (<1%) is a parathyroid carcinoma. the only curative treatment for PHPT is the complete surgical resection of abnormal “hyperfunctioning” tissue. The dosage of PTH intraoperatively is of fundamental relevance confirming the completeness in the removal hyperfunctioning tissue. In only 5% of patients a second surgery is needed. It’s necessary to distinguish: persistent hyperparathyroidism (development of high calcium and PTH values within 6 months of parathyroidectomy for incomplete resection) and recurrent hyperparathyroidism (development of high calcium and PTH values after 6 months of eucalcemia). The most frequent causes of recurrent hyperparathyroidism are: parathyroid adenoma (typical/atypical, single/double), parathyroid carcinoma and a very rare condition known as parathyromatosis, due to benign hyperfunctioning parathyroid tissue scattered in the neck and/or superior mediastinum. Two forms can be distinguished: 1)from hyperplasia of parathyroid during embryologic development (primary form, without previous surgery); 2) from tissue spillage during parathyroidectomy and implantation (secondary form) Primary parathyromatosis may occur in patients with multiple endocrine neoplasia (usually MEN-1), due to parathyroid hyperplasia of embryological residues. The development of scattered rests of benign parathyroid cells can be favored by genetic mutations. The secondary form is more frequent in patients with chronic renal and in patient with hereditary syndromes. To date only 63 cases have been described in literature. In our patient parathyromatosis was due to a combination of genetic alterations (MEN1 gene and CASR gene) never described in the literature. Being a rare syndrome the challenges concern not only the management but also the diagnosis. The main differential diagnosis is parathyroid carcinoma. The intraoperative findings and the histopathologic features are important for making the appropriate diagnosis. Parathyromatosis has high failure rates both with medical and surgical management. Bisphosphonates and cinacalcet therapies are of little benefit and patients often remain symptomatic (5–7). In literature there are some case reports where the addition of denosumab, a humanized monoclonal antibody which inhibits receptor activator of nuclear factor κ-B ligand, can be effective in the treatment of refractory hypercalcemia in parathyroid cancer and in the setting of benign hyperparathyroid-related hypercalcemia such as parathyromatosis (8–10). In patients with MEN1 syndrome, parathyroid tumors (hyperplasia or single or multiple adenomas of the parathyroid glands) express somatostatin receptors (SST) on their cell surface, which can be targeted by somatostatin analogs for tumor localization (^68^Ga-DOTATATE positrion-emission tomography/computed tomography) and therapy. Somatostatin analogues (SSA) control hormone excess and block tumor growth in several type of neuroendocrine tumors (NET). In MEN1 patients SSA are for duodeno-pancreatic NET and pituitary adenomas. Data on the effects of SSA in PHPT are discordant (11, 12).

## Case report

A 52-years-old woman, who had at 36 years old a diagnosis of osteoporosis secondary to primary hyperparathyroidism, underwent surgery with the removal of inferior right parathyroid gland. On histology it was a parathyroid adenoma. She had a family history of osteoporosis and renal stones (mother). After this, she has been followed by an endocrine specialist with referred normal PTH and calcium value. After 10 years she had a recurrence of hyperparathyroidism with PTH 504 pg/ml (12-65), calcium 11,9 mg/dl (8,8–10,6) and phosphorus 1,1 mg/dl (2,5–4,5). The Tc99MIBI scan revealed two foci of increased uptake in the lower pole of the right thyroid lobe and paratracheal left region. Therefore she was referred to our hospital, where she was screened for MEN1, MEN2A and familial benign hypocalciuric hypercalcemia (FHH) because of the youth at diagnosis of osteoporosis. She was on bisphosphonates and vitamin D supplements. Urinary calcium/24h was 148 (50-400), phosphaturia/24h 448 (330-1200), chromogranin A 8 (2-18); negative urinary catecholamine; gastrin 30 (28-185), calcitonin 2 (<11.5), prolactin 7.9 (1.9-25). Genetic screening was done for: FHH heterozygous in exon 8 of the CASR gene of the sequence variant c.2549C>G p.Ala850Gly; MEN1: no evidence of known pathogenetic variants and no genomic rearrangements, in intron 4 variant c.655-6C>A in heterozygosis. HPT-JT: in the CDC73 gene no recognized variants were highlighted as pathogenic (see **Table 1**). Serum calcium and PTH increased in the following years (Ca 10,8 –11,8 and 12 mg/dl and PTH 732–1413 and 1753 pg/ml), with the onset of nephrocalcinosis. Cinacalcet 30 mg was prescribed and, after Tc99MIBI scan and ultrasound, she underwent again surgery with evidence of two adenoma of left and right parathyroid gland. During the operation PTH was monitored with a reduction > 50% after 15-20 minutes from the removal of abnormal tissue but after a month PTH value was 647 pg/ml, serum calcium dropped to 9,3 mg/dl and phosphorus 1,1 md/dl. After three months an ultrasound showed a nodule behind the right thyroid lobe and a PTH-wash was done (> 3278 pg/ml). There was a worsening in the bone mineral density (L1-L4 spine T-score of -3) and hydronephrosis of the right kidney. Serum calcium was 12,8 mg/dl, phosphorus 1,3 mg/dl and PTH 1121 pg/ml, Vitamin D 11,7 ng/ml. She was in therapy with cinacalcet 60 mg/die and vitamin D supplements. On the CT scan, multiple subcentimetric hypervascularized lesions were noted behind the right thyroid lobe and scattered throughout the superior mediastinum, ascribed to parathyroid glands.

**Table 1:** Calcium and PTH values, therapy and other informations throughout the years.

Years	Calcium (mg/dl)	PTH (pg/ml)	Therapy	Other informations
2000	11.6	602	Parathyroidectomy	
2001	9.2	143	none	
2010	11.9	504	Bisphosphonates + vitamin D	
2012	11.1	508	Same above	
2014	12.0	1753	bisphosphonates + vitamin D + cinacalcet 30 mg and after few months parathyroidectomy	
2.2015	9.3	647	Same above	
6.2015	12.8	1121	Bisphosphonates + vitamin D + cinacalcet 60 mg and parathyroidectomy	hydronephrosis of the right kidney
2016	11.0	1098	Same above	TC: Stable the size of the multiple subcentric hypervascular formations in the superior mediastinum
3.2018	11.6	1699	Lanreotide 60 mg i.m/months + cinacalcet 30 mg and vitamin D	PET/TC ^68^Ga-DOTATATE : presence of moderate receptor expression (SSTR2-5) in the right paratracheal site
4.2018	10.3	1394	Lanreotide 120 mg/3 weeks + cinacalcet 30 mg + vitamin D	
6.2018	9.8	700	Same above	Bone mineral density: femoral t-score -3,4
12.2018	11.3	1335	same above; stop lanreotide	Chronic renal failure (stage IIIB)
2019	10.1	1505	Same above	Neck ultrasound: In the right paratracheal area, four hypoechoic areas can be seen, with blending blurred margins measuring 12x11 mm, 8x6 mm and 5x5 mm and 8x9 mm
2021	10.4	1338	Same above	
2022	10.8	1388	Bisphosphonates + vitamin D + cinacalcet 60 mg	

**Figure 1:**
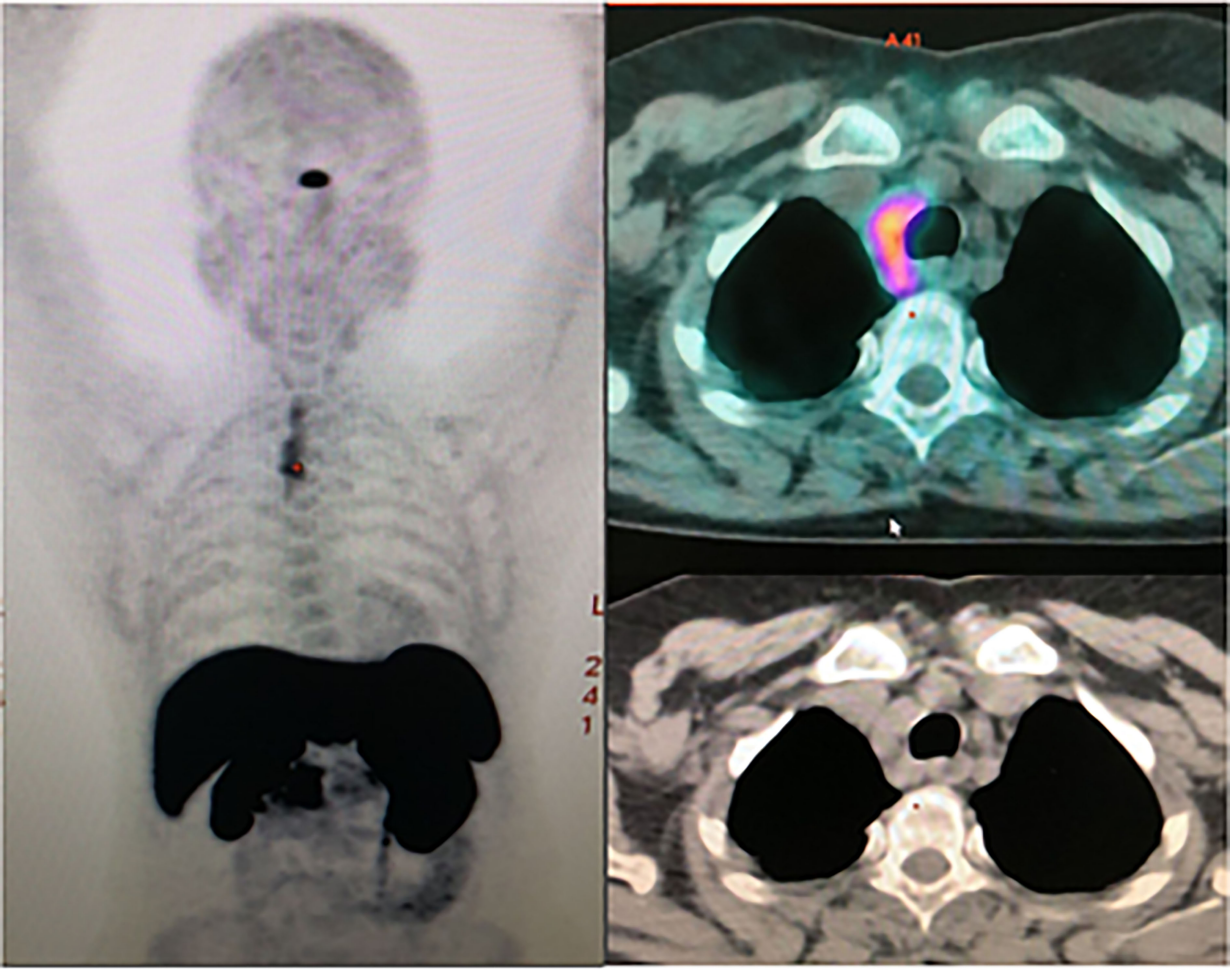
^68^Ga-DOTATATE PET/CT scan: evidence of extended uptake in the neck in then upper mediastinum.

She underwent further surgery with thyroidectomy, on histology were multiple iperplastic parathyroid tissue between the muscle (see **Figure 1**). After a month, PTH was 1251 pg/ml with low levels of vitamin D (3 ng/ml). On the Tc99MIBI scan, there was a recurrence of parathyroid tissue on para-jugular right side. At subsequent follow-up visit PTH was 1098 pg/ml, Vitamin D 15 ng/ml, calcium 11 mg/dl, phosphorus 1.1 mg/dl; albumin 4.5 g/dl, calcitonin 0.5 pg/ml, creatinine 1.2 mg/dl on treatment with Cinacelcet 30 mg twice daily and Vitamin D3 25000 every 15 days. For hydronephrosis an ureteral stent was implanted and to date it is still in follow-up for the deterioration of the right kidney function. The ^68^Ga-DOTATATE PET/CT scan showed an increased uptake in the neck and in the upper mediastinum (**Figure 1**). Lanreotide therapy was given at a dose of 60 mg i.m./4 weeks in addition to cinacalcet 30 mg and vitamin D 25000 UI every 15 days. After two months PTH was 700 pg/ml, calcium 9.8 mg/dl and phosphorus 3 mg/dl.

Unfortunately, after about 6 months, the patient showed a new worsening of the clinical and biochemical parameters.

**Figure 2:**
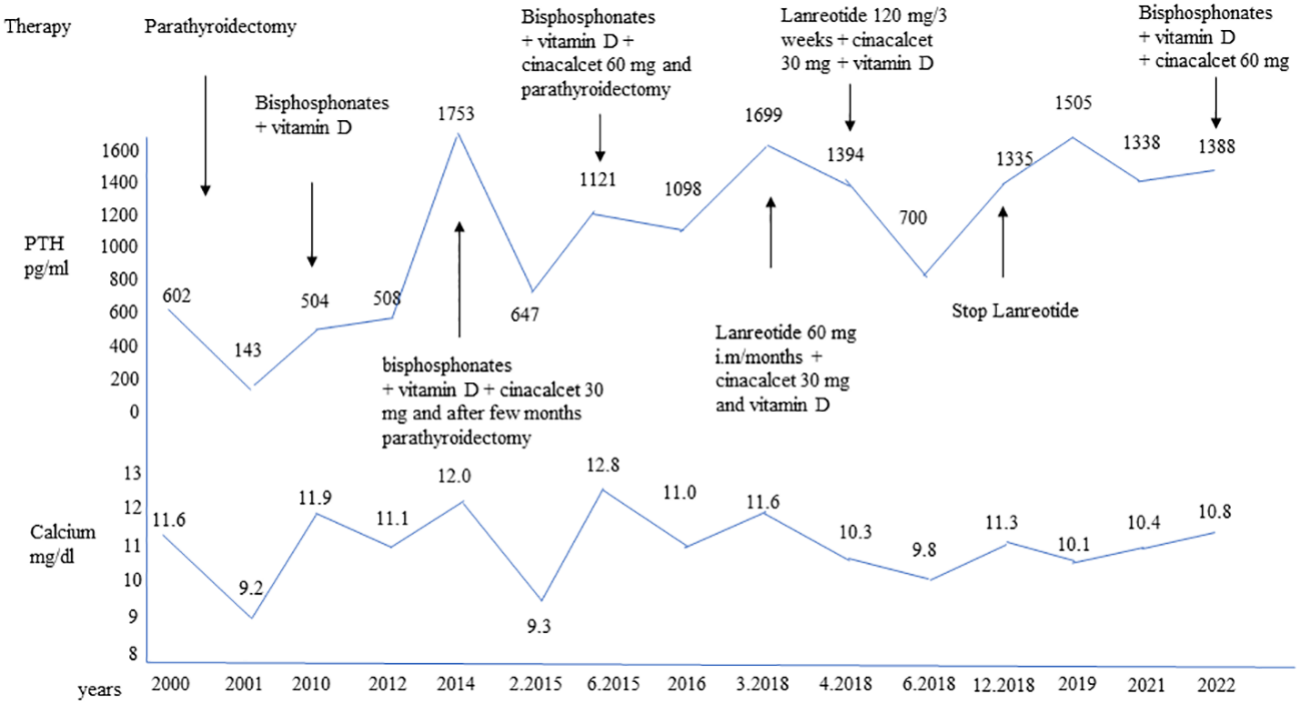
Trend of calcium and PHT values and their respective therapies.

In **Figure 2** the trend of calcium and PTH values and their respective therapies are shown. Furthermore a table shows Calcium and PTH values, therapy and other information throughout the years.

## Discussion

Parathyromatosis is defined as multiple nodules of benign hyperfunctioning parathyroid tissue scattered in the neck and superior mediastinum, resulting in recurrent or persistent hyperparathyroidism (5, 7). The first case of parathyromatosis was described by Palmer et al. parathyromatosis in 1975 in a series of 250 patients with recurrence after parathyroidectomies (13). After 2 years, Reddick classified parathyromatosis as a disease entity (14). Sporadic case reports and small series were described in the literature (overall 63 cases). The most common form of parathyromatosis is the second one in patient with previous neck surgery. The spilling of parathyroid tissue in the neck and/or mediastinum during surgery for adenoma or hyperplastic tissue can lead to a hyperplasia of these tissue implants (mainly due tohypocalcemia, hypophosphatemia and hypovitaminosis D for chronic renal failure). The primary form due to embryonic parathyroid residues is less common. Patients with multiple endocrine neoplasia (usually MEN-1) can develop the primary form of parathyromatosis. Genetic mutations could favor the development of scattered rests of benign parathyroid cells. The secondary form is more frequent in patients with chronic renal and in patient with hereditary syndromes (15). Symptoms include fatigue, weakness, polydipsia and polyuria, bone and back pain, constipation, mood disorders, loss of appetite with weight loss and bone fracture. Clinically, moderate hypercalcemia(usually less than 14 mg/dl) is present. Serum PTH levels decrease after surgery and then increase again. Hypophosphatemia and high alkaline phophatase value may be present. The diagnosis should be suspected if previous parathyroid surgeries and/or recurrent/persistent high PTH levels (5).Hage et al. (16) showed how the initial diagnosis of benign parathyroid lesion leads to repeat surgeries for hyperplasia, adenoma or ectopic parathyroid tissue. Multiple local recurrences after parathyroidectomy could lead to suspect the diagnosis of parathyroid carcinoma, so pathological distinction is crucial for treatment. During surgery, in parathyromatosis multiple small grey nodules are typical, instead in the parathyroid carcinoma as a solitary large hard white nodule with infiltrative borders is more frequent. In both cases adherent fibrous tissue is present. Histological features associated with malignancy are: capsular and/or vascular invasion, 5/10 HPF mitoses, tumor necrosis, cellular atypia, trabecular growth pattern andintratumoral fibrosis. However, these histological features don’t absolutely distinguish the two conditions. Use of some immunohistochemical markers (the overexpression of galectin-3 and the loss of parafibromin and Rb), could be used for distinction of benignant of malignant form. Instead the difference between parathyromatosis and parathyroid adenoma is the lack of the classical features and the encapsulation (5, 6). Parathyromatosis’s management remains challenging. The aim of surgery is the removal of all the disseminated nodules but unfortunately it is in almost all of cases unsuccessful so medical management is crucial. Vitamin D therapy leads to PTH level and bone turnover reduction. Cinacalcet, an oral calciomimetic that decrease PTH secretion binding calcium sensing receptor, has been used with success mainly in chronic renal failure patients. Another group of drug with contrastant and often inconsistent results in primary hyperparathyroidism are the bisphosphonates (17). Since our patient had a gene mutation associated with MEN 1, we performed a ^68^Ga-DOTATATE PET/CT scan to see if parathyroid cells express somatostatin receptors (11). Having obtained a good response to ^68^Ga-DOTATATE positron-emission tomography/computed tomography, we started the somatostatin analogue therapy (SSA) (Lanreotide 120 mg, i.m. every 4 weeks). After two months of therapy, PTH and calcium levels decreased but did not normalize. Several studies in literature reported the use of somatostatin analogs in the treatment of primitive hyperparapathyroidism in patients with MEN 1 with conflicting results. SSA are used in gastroentero- pancreatic neuroendocrine tumors and/or pituitary adenomas, because they express SST receptors. Also parathyroid adenomas are included in the NET; in fact in patients with MEN1, they showed a common pathogenic origin with gastro-entero-pancreatic NET and pituitary adenomas. A study by Faggiano et al. showed the potential utility of long-acting somatostatin analogues in hyperparathyroidism in patients with MEN-1 (12). In fact, during a 6 month therapy with depot somatostatin analogue, serum PTH, calcium and phosphorus values, urine calcium excretion and phosphorus renal reabsorption resulted stably improved Octreotide could lead to an improvement of calcium and phosphorus values through direct effects on PTH hypersecretion, due to SST expression on parathyroid cells.

## Review of literature

To our knowledge 63 cases of parathyromatosis have previously been described (**Table 2**).

**Table 2:** Parathyromatosis: review of literature.

First author and Year (Ref.)	Cases (n)	Treatment	Outcome
Palmer et al., 1975 (13)	2	1/1 reoperation	Remission; persistence
Reddick et al., 1977 (14)	1	3 reoperations	Remission
Rattner et al., 1985 (18)	2	1/1/1/3 reoperations	All remission
Akerstrom et al., 1988 (19)	3	1/2/2 reoperations	Remission
Fitko et al., 1990 (20)	1	4 reoperations	Death
Sokol et al., 1993 (21)	1	2 reoperations	Persistence
Kollmorgen et al., 1994 (22)	1	1 reoperation	Remission
Stehman-Breen et al., 1996 (23)	5	2 reoperations; foream amputation; 3 reoperations; 5 reoperations, 1 reoperation	Death; foream amputation, Persistence; Remission; Death
Lee et al., 2001 (24)	1	2 reoperations	Persistence
Baloch et al., 2001 (15)	1	1 reoperation	Remission
Lentsch et al 2003 (25)	1	1 reoperation	Remission
Evans et al., 2005 (26)	1	1 reoperation	Remission
Daphnis et al., 2006 (27)	1	1 reoperation + cinacalcet	Mild remission
Tublin et al., 2007 (28)	1	2 reoperations	Remission
Unbehaun et al., 2007 (29)	1	1 reoperation + cinacalcet	Remission
Fernandez-Ranvier et al., 2007 (5)	13	1 reoperation	9 Persistent; 4 remission
Vulpio et al., 2011 (30)	1	2 reoperation + cinacalcet	Remission
Diaconescu et al., 2011 (31)	1	1 reoperation	Remission
Sim et al., 2012 (32)	1	2 reoperations + cinacalcet+zoledronic acid	Mild remission
Wu et al., 2012 (33)	1	1 reoperation	Remission
Mohammadi et al., 2012 (34)	1	1 reoperation	Remission
Hage et al., 2012 (16)	1	4 reoperations + cinacalcet	Mild remission
Pinnamaneni et al., 2013 (35)	1	2 reoperations	Persistence
Twigt et al., 2013 (36)	2	1/3 reoperations	Remission
Edling et al., 2014 (37)	1	1 reoperation + cinacalcet	Remission
Scorza et al., 2014 (7)	1	3 reoperations + cinacalcet	Persistence
Sharma et al., 2016 (38)	1	2 reoperations	Remission
Oueslati et al., 2016 (39)	1	2 reoperations + cinacalcet	Remission
Jain et al., 2017 (17)	1	2 reoperations	Remission
Achour et al., 2017 (40)	1	1 reoperation	Remission
Nakamura et al., 2017 (41)	1	1 reoperation + cinacalcet	Remission
Aggarwal et al., 2017 (42)	1	1 reoperation	Remission
Bartoňová et al., 2018 (43)	1	1 reoperation	Remission
Wei et al., 2019 (44)	1	2 reoperations + cinacalcet	Persistence
Cao et al., 2019 (45)	1	2 reoperations	Remission
Miller et al., 2019 (46)	1	1 reoperation	Data not available
Haciyanli et al., 2019 (47)	1	2 reoperations	Remission
Altin et al., 2020 (48)	1	1 operation	Remission
Yang et al., 2020 (49)	1	1 reoperation	Remission
Ilyicheva et al 2021 (50)	1	1 reoperation	Remission
Tzotzas et al., 2022 (10)	1	1 reoperation + cinacalcet	Remission
Latgé et al 2022 (51)	1	2 reoperations	Remission
Total cases: 63			

In most cases in the literature (42 cases) the disease went into remission. In 16 cases, however, parathyromatosis remained persistent and in 3 cases it led to death from complications related to hyperparathyroidism.

In 9 cases, cinacalcet was used in addition to surgical treatment. In 2 of these cases the disease remained in the persistent form.

## Conclusion

Parathyromatosis is a rare cause of recurrent or persistent hyperparathyroidism characterized by the presence of multiple parathyroid tissue foci in the neck and mediastinum. It usually occurs in patients after parathyroidectomy for adenoma or hyperplastic disease (secondary form), but may occur “*de novo*” (primary form). Only about 60 cases have been described to date in the literature, in patients with familial forms of primary hyperparathyroidism (MEN1, FIHPT) or with chronic kidney disease. The main differential diagnosis is with parathyroid carcinoma. It is a difficult condition to diagnose and to treat radically. In patients with MEN1 syndrome, parathyroid tumors express somatostatin receptors on their cell surface, which can be targeted by somatostatin analogues for both tumor localization (^68^Ga-DOTATATE PET/CT) and treatment.

We report a rare case of parathyromatosis due to a combination of genetic alterations (MEN1 gene and CASR gene) never described in the literature. The lack of complete removal of parathyroid tissue foci, despite repeated surgery and medical treatment caused metabolic consequences of chronic hypercalcemia. Lanreotide treatment was beneficial in our patient, but due to the rarity of this condition, his potential aggressiveness of and the lack of clinical evidence in large series medical treatment need to be personalized.

An important challenge includes the functional relevance of rare genetic variants and/or the i of mutations in non-coding regions, the possible influence of epigenetic mechanisms in this rare condition which can help to identify potential new therapies.

## Author contributions

Conception and design, GS, MG, and GP. Provision of study materials or patients, GS, MG, and GP. Collection and assembly of data, GS, MG, DA, RM, MT, MR, and GP. Data analysis and interpretation, GS, MG, and GP. Manuscript writing, GS, MG, and GP. Revision and final approval of manuscript, all authors. All authors contributed to the article and approved the submitted version.
